# The experience of leaving a valuable object: An investigation of emotional processes related to Hoarding disorder features

**DOI:** 10.1371/journal.pone.0280933

**Published:** 2023-02-14

**Authors:** Susanna Pardini, Silvia Olivetto, Francesca Fusina, Caterina Novara

**Affiliations:** 1 Department of General Psychology, University of Padova, Padova, Italy; 2 Digital Health Research Unit, Centre for Health and Wellbeing, Fondazione Bruno Kessler, Trento, Italy; 3 Human Inspired Technology Research Centre (HIT), University of Padova, Padova, Italy; 4 Padova Neuroscience Center, University of Padova, Padova, Italy; Murcia University, Spain, SPAIN

## Abstract

One of the core features of hoarding is a significant resistance to discarding objects, which is fueled by dysfunctional beliefs and unwarranted negative emotions that hoarders tend to feel when disposing of their possessions. To our knowledge, longitudinal studies investigating the psychological effects that people who hoard experience after separating from their valuable possessions have yet to be conducted. Our study’s principal aim was to explore psychological processes that individuals with high hoarding features (n = 53; 49.1%) and individuals with low hoarding features (n = 55; 50.9%) experienced when they had to separate from a valuable possession. To do this, we evaluated participants’ thoughts and feelings at several time points after they had to leave a valuable object at the University laboratory (evaluations were specifically conducted at baseline, during the week, and at the end of the week). To investigate hoarding and anxiety, as well as depressive, obsessive-compulsive, and emotional processes-related features, a mixed-method approach was employed involving self-report questionnaires, ad hoc surveys, and a daily self-monitoring schedule. Our findings showed that compared to participants with low hoarding features, participants with high hoarding traits: 1) scored higher for anxiety sensitivity, distress tolerance, and emotional dysregulation; 2) reported having more negative emotions when leaving their object; 3) had more intrusive object-related beliefs; and 4) experienced a higher frequency of negative emotions as well as a higher level of distress during the week. Both groups experienced more negative emotions in the first part of the week, which decreased as the time at which participants could receive their object back drew closer. Finally, dysfunctional beliefs about leaving a personal object (Saving Cognitions Inventory), sensitivity to anxiety (Anxiety Sensitivity Index-3), and distress tolerance (Distress Tolerance Scale) contributed to the level of discomfort that participants with higher hoarding scores reported when they had to leave their possession. These results highlight the relevance of emotional processes in the hoarding disorder framework as well as underscore the importance of assessing and treating them in clinical settings.

## Introduction

Hoarding disorder (HD) is a psychological problem characterized by difficulties in discarding objects, the need to save newly acquired items, and the accumulation of possessions [1]. These behaviors negatively impact the daily functioning of people who hoard (PwH) and the people that live with them. Hoarding is fairly widespread, with a pooled estimated prevalence of 2.5% [2]. Therefore, improving assessment and treatment for HD is crucial.

The current gold standard treatment for HD is based on the cognitive and behavioral model [3, 4] which has been shown to be effective in decreasing HD symptoms. Other treatments seem to only result in low-to-moderate clinically significant improvements and are limited to the clutter domain [5].

The HD cognitive and behavioral model considers several variables, including vulnerability factors (e.g., traumatic events, mood and anxiety disorders, difficulties in cognitive functions) and one’s beliefs and feelings related to their possessions. These elements contribute to the development and maintenance of HD.

People who hoard (PwH) also exhibit dysfunctional beliefs, such as an exaggerated sense of responsibility or attachment to their possessions. Although people with HD and people without a hoarding problem commonly experience negative emotions of similar intensity levels when discarding a valuable object, the former group has the tendency to unnecessarily save items in an attempt to avoid these negative feelings [6]. Therefore, the fact that most people have similar negative emotions but only some display hoarding behaviors shows that the ability to understand and regulate one’s negative emotions as well as a tendency to attribute greater value to a greater number of items plays a role in HD [6].

Hoarding disorder is also characterized by transdiagnostic constructs that have the potential to influence the presence and intensity of typical hoarding symptoms. Transdiagnostic constructs that are particularly relevant to HD include difficulties in tolerating distress, a decreased ability to regulate emotions and increased emotional reactivity and anxiety sensitivity [6–19].

The assessment of these transdiagnostic constructs is essential, since they allow therapists and researchers to understand how general dysfunctional processes interact with the HD core features and their roles in facilitating and maintaining hoarding symptomatology (e.g., [7]). Since difficulties in tolerating fear and discomfort is a maintenance factor of HD and associated behaviors (such as to avoid the act of throwing away possessions), the management of these processes should be integrated into HD treatment and thoroughly investigated during the act of discarding [8–11, 13, 15, 17, 20–23].

To the best of our knowledge, Frost and colleague [24] are among the few to have examined the behavioral, emotional, and cognitive processes that are related to discarding behaviors in an HD group and in a non-HD group. Frost et al. demonstrated that individuals with HD experienced greater levels and durations of distress, negative affect, and maladaptive beliefs when they needed to decide if they would throw away a possession or a new object as compared with a non-HD group. In the current study, we have assessed emotional regulation constructs in individuals with High (H)-HD and individuals with Low (L)-HD features that were asked to leave an important possession in the university laboratory. We also monitored emotional, cognitive, and behavioral components based on a daily self-monitoring schedule that participants filled out for seven days.

The present study aimed to investigate the relationships between HD symptoms, distress tolerance, emotional dysregulation, dysfunctional beliefs, and negative emotions. We also aimed to explore the type and frequency of thoughts and feelings that participants expressed throughout the week after leaving a possession. In accordance with the literature, we expected to find higher scores in the H-HD group than in the L-HD group on questionnaires investigating distress tolerance, emotional dysregulation, and dysfunctional beliefs. Regarding longitudinal data, we expected to find:

- Greater negative emotions experienced in the H-HD group compared to the L-HD group when participants left their object at the laboratory (T1);
- More negative emotions reported during the T1 assessment compared to when participants’ objects were returned to them (T2), especially in those with H-HD scores;
- A higher frequency of thoughts and feelings expressed during the week by the H-HD group compared to the L-HD group;
- An impact of HD-related dysfunctional thoughts, difficulties in tolerating distress, and emotional dysregulation on the frequency of thoughts and feelings experienced during the week. We expected these variables to have an impact on the intensity and discomfort that participants reported about leaving their possession at the laboratory.

## Methods

### Participants

In the first phase of the study, we recruited 1410 individuals from the general population. Participants came from Northern Italy and were recruited with a snowball sampling method. To participate in the research, individuals had to give their written informed consent providing accurate self-reported information. Inclusion criteria required participants to be native Italian speakers, 18 years or older, without a documented diagnosis of severe mental, neurological, or linguistic disorders. Moreover, individuals had to have an important personal object, regardless of its actual value [1].

Since our sample comprised individuals from the general population without a hoarding diagnosis, to maximize any differences related to the investigated constructs, only those who had a score greater than the 90th percentile and lower than the 10th percentile at the Saving Inventory-Revised (SI-R) were included for participation in the subsequent phases of the research. Cut-offs of the SI-R were derived from data on an Italian sample [25], and individuals were divided into two groups named H-HD (n = 53; 49.1%), including those who scored equal or higher than 37 on the SI-R (corresponding to the 90th percentile based on the total sample scores), and L-HD (n = 55; 50.9%), with a SI-R total raw score equal or lower than 5 (corresponding to the 10th percentile based on the total sample scores).

Considering the scores our sample obtained as compared with clinical cut-offs for hoarding, individuals in the H-HD group obtained a mean score of 44.17 (SD = 9.24; range: 37–78), which is considered in the range of clinically significant hoarding features (the clinical cut-off of the SI-R in an Italian sample was found to be at or above 37 [25]; based on a recent non-Italian study, the SI-R Total cut-off score is 39 [26]).

On the other hand, individuals in the L-HD group had a mean score of 3.13 (SD = 1.53; range: 0–5). In the literature, the average score for people with hoarding is 62 (SD = 12.7) and for people without HD is 23.7 (SD = 13.2). However, it is to be noted, that more than SI-R scores alone are not enough to enable the diagnosis of full-fledged hoarding in our sample; indeed, the questionnaire only refers to difficulties in getting rid of objects. Additionally, our subjects did not report any significant clutter or actual hoarding of possessions in their homes.

**Fig 1:**
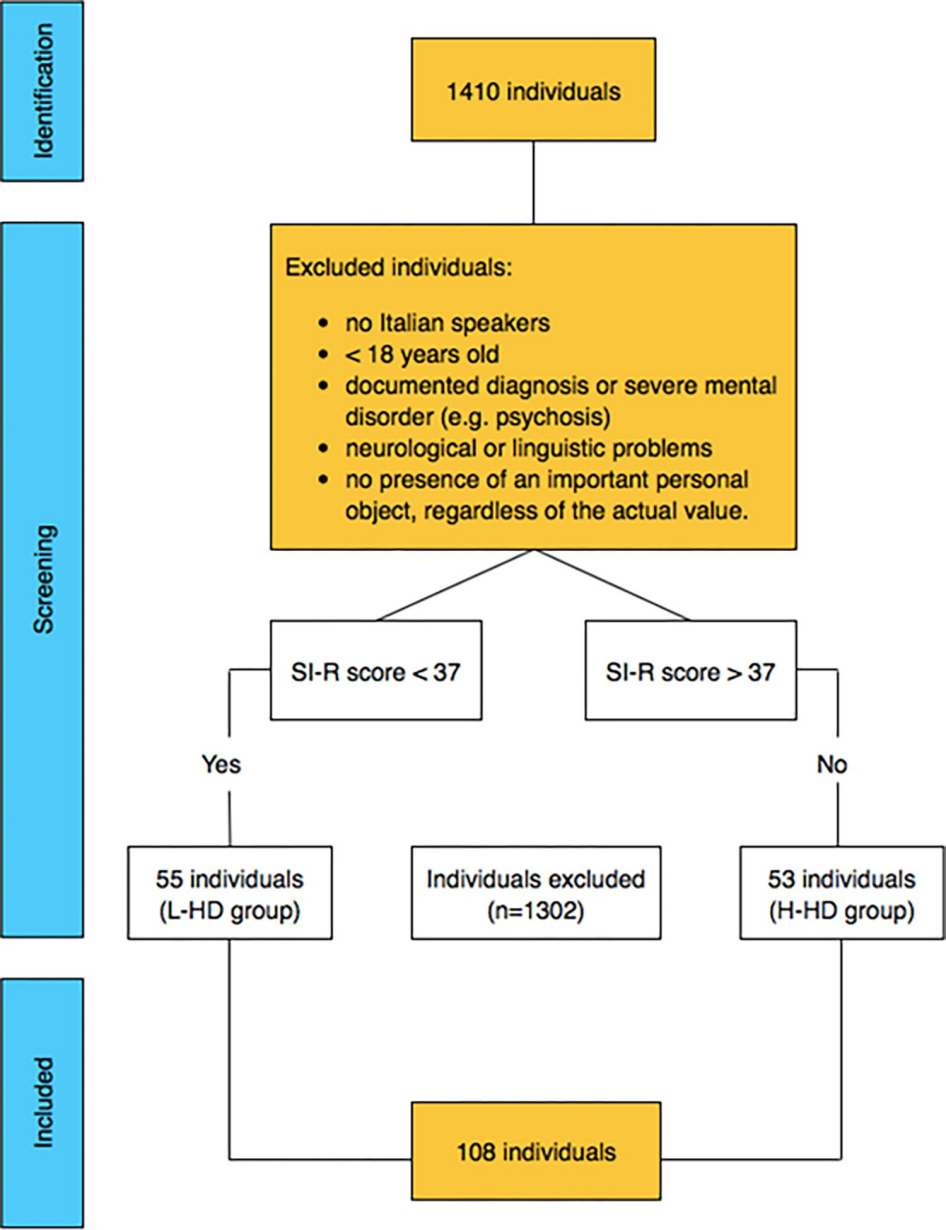
Flow chart.

The final sample comprised 108 adults, who were mainly University students (n = 74; 68.5%) (Fig 1).

### Procedures

Before the recruitment phase, a pilot testing procedure was carried out, and consisted in administering the entire protocol to a convenience sample of 10 individuals from the general population.

Individuals participated voluntarily and gave their written informed consent before taking part in the study. They participated in three sessions that lasted 70–80 minutes globally. Each measure was administered in counterbalanced order to avoid any biased effects. Four Psychology Master’s Degree students administered the experiment under supervision.

This study was conducted in accordance with the Declaration of Helsinki and approved by the Ethics Committee of the Department of General Psychology at the University of Padua (protocol number: 2288). Informed consent was obtained for experimentation with human subjects.

#### T0

To appropriately select the H-HD and the L-HD groups, participants filled out the SI-R, a Demographic Schedule, and two open-ended questions to understand if they had important objects (assessed on a scale from 0 to 100) and, if so, why they saved them.

Then, those that were deemed eligible for the study were contacted by email and asked to bring their valuable possession to the laboratory. Personal objects were grouped into eight categories (clothes, photographs, keyrings, diaries/books, lucky charms, cups, rings, toys) and, from an observational perspective about the type of items, no differences have been shown between those carried by the H-HD and the L-HD groups.

Participants did not receive any compensation for participating in the study.

#### T1

The second step took place one week after the T0 phase, at the Experimental Psychopathology Laboratory at the Department of General Psychology, University of Padova. First, individuals were told for the first time to leave their objects for a week; participants were told that we would lock away their objects untouched for the entire week. If they accepted, individuals had to fill out another written consent form to confirm their participation in the subsequent phases. All eligible individuals accepted to take part in the entire experimental procedure. They filled out the Saving Cognition Inventory (SCI), the Obsessive-Compulsive Inventory-Revised (OCI-R), the Beck Anxiety Inventory (BAI), the Beck Depression Inventory second edition (BDI-II), the Anxiety Sensitivity Index-3 (ASI-3), the Distress Tolerance Scale (DTS), the Difficulties in Emotion Regulation Scale (DERS), the Pers Emotional Reactivity Scale (PERS), the Positive and Negative Affective Schedule (PANAS), and the State-Trait Anxiety Inventory-Y (STAI-Y). Then, participants answered questions related to the objects included in a Qualitative Survey.

#### During the week

Participants were instructed to complete a daily self-monitoring schedule to investigate thoughts (e.g., “I would like to have my object now”), type of feelings (e.g., worry, anger, sadness), intensity of emotions (from 0 to 100), and level of distress related to leaving the objects in the laboratory (from 0 to 100). Specifically, participants had to complete their schedules in a.docx file and share it with the research team by email with smartphones or computer devices at the end of each day (before going to sleep). To avoid participant forgetfulness, the experimenters sent them daily email reminders around 6 pm.

### T2

At the end of the week, individuals returned to the laboratory to retrieve their objects and fill out only the questionnaires that investigated state constructs (the PANAS and the STAI-Y). Based on the same Qualitative Survey administered in T1, participants had to express what type of emotions, and the respective intensity, they felt in taking back their object. Finally, the objects were returned, and individuals had to sign the Quality Survey to confirm they received them.

### Measures

Data about socio-demographic features have been assessed based on a **Demographic schedule.**

An **Ad Hoc Survey**, specifically developed for the present research, was administered during the assessment in T1 to ask if participants lived alone or with other people, what was the object they left in the laboratory, and if there were other valuable items they decided not to bring. Additionally, individuals had to evaluate how important their object was (based on a scale of 0 to 100) and why it was essential. At the end of the T1 assessment, participants had to express the intensity of the distress they felt in leaving their objects and the type of emotions they went through (from 0 to 100). The information on the importance and distress intensity were evaluated with a **Visual Analogue Scale (VAS)** from 0 to 100, inspired by other studies investigating emotions and other features related to HD [27].

The last part of this measure was used in T2 to assess the type and intensity of participants’ emotions (from 0 to 100) related to having taken back their items.

The type and frequency of thoughts and feelings, as well as the distress level related to the object left in the laboratory, were recorded by asking participants to fill out a **Daily self-monitoring schedule.** This measure was inspired by the information included in the assessment part of the “Clutter Visualization Exercise” [28].

Hoarding features were assessed based on the **Saving Inventory-Revised (SI-R)**, and the **Saving Cognition Inventory (SCI)**. The SI-R [29, 30] comprises 23 items assessing HD core features. Items are divided into three subscales corresponding to HD’s three main domains: "Difficulty in Discarding", "Excessive Acquisition", and "Clutter". Scores are assigned on a 5-point Likert Scale ranging from 0 to 4 (0 = not at all; 4 = extreme). It also emphasizes good convergent validity, highlighted by positive correlations with other hoarding scales. The original version shows a good internal consistency (Cronbach’s α = .94) for each subscale (α ranged from .80 to .93). Good test-retest reliability also emerged (r = 0.96). The validation of the Italian version of the SI-R also shows a good internal consistency both in the total score (α = .88) and in the subscales (.79<α < .83). In our sample, we noticed a good internal consistency for the SI-R total score (α = .97) and subscales (.93<α < .97). The SCI [25, 31] is a 23-items questionnaire divided into four subscales that investigate dysfunctional beliefs related to situations implying imagining or actually discarding objects (“Emotional Attachment”, “Control”, “Responsibility” and “Memory”). The score is based on a 7-point Likert scale (1 = not at all; 7 = very much). The original version evidenced high internal consistency (total score: α = .96; subscales: .86<α < .95), as well as good convergent validity (.60<r < .80). The Italian version has confirmed a good internal consistency for the total and subscales (.69<α < .92), and an acceptable between-scale intercorrelation (.44<r < .72). A good-to-moderate convergent validity based on the correlations between SI-R and SCI emerged (.29<r < .65). In our sample, we noticed an excellent internal consistency for the SCI total score (α = .95) and subscales (.81<α < .92).

To investigate the other related constructs such as obsessive-compulsive, anxiety, and depressive symptoms, the following self-report questionnaires have been used: 1) the **Obsessive-Compulsive Inventory-Revised** (OCI-R) [32, 33], an 18-items test assessing the severity of OC symptoms on a 5-point Likert scale. The items are grouped into six subscales: "Washing", "Checking", "Ordering", "Obsessing", "Hoarding", and "Mental Neutralizing", composing an additional final total score. The original version has good reliability, validity, and good test-retest stability [32]. The Italian version of the OCI-R [33] showed good internal consistency and 30-day retest reliability ranging from 0.76 to 0.99, and good convergent, divergent, and criterion validity [32]. In our sample, we noticed good internal consistency (α = .90) in each subscale (.61<α < .90); 2) the **Beck Anxiety Inventory** (BAI) [34–36], a 21-items measure assessing the severity of anxiety symptoms, with a four-point Likert scale ranging from 0 to 3 (0 = not at all; 3 = severely—it bothered me a lot). It shows an excellent internal consistency (α = .92) and a good test-retest reliability over one week (r = .75). The Italian version’s internal consistency was good (α = .90). The current study showed good internal consistency (α = .91); 3) the **Beck Depression Inventory-Second Edition** (BDI-II) [37, 38], a 21-items test used to assess the severity of depression symptoms considering the two weeks preceding the evaluation. It is based on a four-point Likert scale (from 0 to 4; 0 corresponds to the absence of the symptom/feeling and 4 to its entire presence). The original version showed excellent internal consistency in a sample of university students (α = .93) and a clinical sample (α = .92). The test-retest reliability across a period of one week was good (r = 0.93), as well as convergent and divergent validity. A good convergent validity, based on positive correlations with other depressive symptom questionnaires, has been evidenced (0.71<r<0.84). The Italian version showed a good internal consistency considering a sample of university students, patients with depression, and a group of individuals selected from the general population (.80<α < .87). The test-retest reliability across the period of one month was good (r = 0.76), as well as convergent, divergent, and criterion validity. The current study highlighted a good internal consistency of the questionnaire (α = .89).

The main transdiagnostic features, such as anxiety sensitivity, distress tolerance, emotional reactivity, emotional regulation, state anxiety and valence of emotions, were investigated with the following measures: 1) the **Anxiety Sensitivity Index-3** (ASI-3) [39–41], an 18-items self-report questionnaire evaluated on a 5-point Likert scale from 0 to 4, which refers to the three main domains of anxiety sensitivity: “Physical Concerns” (beliefs and fears regarding one’s physical reactions), “Cognitive Concerns” (fear of losing control) and “Social Concerns” (fear to manifest physical reactions in public). The original and the Italian versions evidenced good internal consistency (α = .92 and α = .90, respectively), and the test has also shown a fair test-retest reliability index (r = 0.76). Based on the study by Ghisi and colleagues [41], the Italian version showed good reliability for the general factor, for test-retest reliability, and adequate convergent and divergent validity. Finally, in our sample, we noticed a good internal consistency for the ASI-3 total score (α = .91) and subscales (.81<α < .90); 2) the **Distress Tolerance Scale** (DTS) [42, 43], a 15-items self-report questionnaire which is divided into four subscales. The DTS investigates the ability to tolerate negative emotional states based on the following constructs: "Tolerance" (ability to tolerate emotional distress), "Absorption" (concerning the reserve of attentional resources used for the negative emotions experienced), "Regulation" (referring to the strategies used for emotional regulation) and "Appraisal" (the subjective evaluation of negative emotional states). Items are rated on a 5-point scale (1 = strongly agree; 5 = strongly disagree). Higher scores correspond to a higher ability in managing distress and negative emotional states. The Italian version highlighted good internal consistency, temporal stability, and construct validity. The internal consistency (α = .89) and test-retest reliability (r = 0.61) are good. In our sample, Cronbach’s α was equal to .92, and we also noticed a good internal consistency of each subscale (.77<α < .85); 3) the **Difficulties in Emotion Regulation Scale** (DERS) [44, 45], a 36-items self-report questionnaire that measures the difficulties in regulating negative emotions44. The items are grouped into 6 scales: “Lack of Acceptance”, “Difficulty in Distraction”, “Lack of Control”, “Lack of Confidence”, “Difficulty in Recognizing Emotions”, and “Reduced Self-Awareness”. The Italian version showed high internal consistency both in the total score (α = .90) and in the six scales (.74<α < .88). In our sample, Cronbach’s α was equal to .95, and in each subscale it ranged from .81 to .93; 4) the **Perth Emotional Reactivity Scale** (PERS) ([46]; ad hoc Italian translation), a 30-items self-report questionnaire used to measure emotional reactivity; specifically, it refers to eight subscales that investigate general reactivity, intensity, duration, and activation both for negative and positive emotional states. The two primary subscales are the "General Negative Reactivity Scale (GNRS)" and the "General Positive Reactivity Scale (GPRS)", to which the other subscales refer. The original version showed that six factors characterize PERS; excellent internal reliability has been highlighted (.81<α < .94) and excellent construct validity. We noticed good internal consistency of PERS in our sample, particularly for each subscale (.81<α < .93); 5) the **Positive and Negative Affect Schedule** (PANAS) [47, 48], a 20-items that measures positive (PA) and negative affect (NA). It is a list of 20 feelings: 10 referring to PA (e.g., "active") and 10 to NA (e.g., "nervous"). Items are evaluated on a scale ranging from 1 to 5 (1 = slightly; 5 = extremely), which refers to how one person feels in a precise moment47. Both versions have good internal consistency (α PA = .89, α NA = .85 in the original version; PAα = .83, NAα = .85 in the Italian version) and fair test-retest reliability (r = 0.54 and r = 0.45 compared to the first and r = 0.65 and r = 0.52 compared to the second); 6) the **State-Trait Anxiety Inventory-Y1** (STAI-Y1) [49, 50], a 20-items questionnaire that measures the level of anxiety experienced at the moment the test is filled out. Scores are given on a Likert scale ranging from 1 to 4. Internal consistency is very high both in the original version (.86<α < .95) and in the Italian one (.91<α < .95). The test-retest reliability was quite good (T1: 0.34<r<0.62; T2: r = 0.49).

## Results

### Data analysis

Anonymized data were processed with SPSS Statistics-Version 22.0 and 27.0 [51, 52].

Cronbach’s alpha was calculated for all scales and subscales of the self-report questionnaires.

Demographic features were described using the means and standard deviations for quantitative data and percentages for categorical data. Multivariate and Univariate ANOVA and a Chi-squared index were conducted to explore differences between groups.

For MANOVAs, the Fisher’s F and Partial Eta Squared were reported as effect sizes.

Repeated-measures MANOVAs were run to investigate the effects of “time” (T1-T2) and “group” (H-HD, L-HD) on the reported state anxiety, negative and positive emotions based on the PANAS and the Daily Self-Monitoring Schedule. T-tests for paired samples have been used to investigate differences regarding state anxiety, and negative and positive emotions for each group.

Descriptive data about thoughts and emotions experienced at T1, T2, and during the week were derived from frequencies. The open-ended questions related to thoughts were analyzed thematically following an inductive, data-driven approach based on the procedure outlined by Braun and Clarke [53]. Two independent coders manually performed sentiment analysis to understand the valence of the emotions that participants felt during the week. The two raters discussed any discrepancies in coding until they met an agreement. Responses indicating positive (e.g.,“*sadness”)*, neutral (e.g., “*nothing in particular”)*, and negative emotions (e.g., *“Sadness”*, *“I missed it”*, *“I’m sad because I don’t have my object with me”*) were scored as 0, 1, and 2 respectively.

Finally, multiple linear regression analyses were performed to investigate if thoughts’ frequency, negative emotions, feeling intensity, and discomfort were related to hoarding, distress tolerance, and emotion regulation.

### Demographic variables and differences between groups

57.7% of the H-HD group were women (n = 41), with a mean age of 28.25 years (SD = 9.27, ranging from 20 to 56 years) and a mean of 16.35 years of education (SD = 2.39; ranging from 9 to 22 years). As far as the L-HD group was concerned, 42.3% were women (n = 30), with a mean age of 26.91 (SD = 9.76; ranging from 21 to 59 years), and a mean of 16.40 years of education (SD = 2.43; ranging from 8 to 20 years). Considering these demographic variables, we found no statistically significant differences between the two groups (Age: Fisher F = 0.53; p-value = .47; Years of Education: Fisher F = 0.01; p-value = .92) except for gender, with a major prevalence of women in the H-HD group (Gender: *χ*^2^ = 6.24; p-value = .01).

A difference between the groups emerged for the "Psychological Problems" variable (*χ*^2^ = 4.85; p-value = .03): more individuals in the H-HD group (n = 22; 64.7%), rather than the L-HD one (n = 12; 35.3%), reported suffering from a psychological problem without a diagnosis (e.g., depressive or anxiety symptoms) (see Table 1).

**Table 1:** Demographic features and comparisons considering psychological constructs.

	H-HD^f^ N^a^ = 53	L-HD^g^ N^a^ = 55	F(1,107)	χ2	Partial Eta squared
**Gender (Women) (%)**	41 (57.7%)	30 (42.3%)		6.24*	0.24
**Age**					
M^b^ (SD)^c^	28.25	26.91 (9.76)	0.53		
Range (min^d^–max^e^)	(9.27) (20–56)	(21–59)			0.005
**School years**			0.01		0.000
M^b^ (SD)^c^	16.35	16.40 (2.43)			
Range (min^d^–max^e^)	(2.39) (9–22)	(8–20)			
**Marital Status**					
**single (%)**	42 (47.7%)	46 (52.3%)		1.40	0.10
**Medication**					
**(%)**	7 (70%)	3 (30%)		1.94	0.14
**Psychological Problems**	22	12			
**(%)**	(64.7%)	(35.3%)		4.85*	0.21
**Objects (Yes)**	36	11			
**(%)**	(76.6%)	(23.4%)		18.79***	0.48
BDI-II^h^	13.9	6.22	22.3***		
M^b^ (DS)^c^	(8.99)	(7.00)			0.18
BAI^i^	19.3	7.22	56.9***		
M^b^ (DS)^c^	(9.16)	(7.35)			0.35
OCI-R^l^	20.7	4.51	102.7***		
M^b^ (DS)^c^	(10.1)	(5.47)			0.50

Notes

^a^N = size of the group

^b^M = Mean

^c^SD = Standard Deviation

^d^min. = Minimum

^e^max. = Maximum

^f^H-HD = High-Hoarding Disorder group

^g^L-HD = Low-Hoarding Disorder group

^h^BDI-II = Beck Depression Inventory-II-total score

^i^BAI = Beck Anxiety Inventory-total score

^l^OCI-R = Obsessive-Compulsive Inventory-Revised-total score

*** = p-value < .001

** = p-value < .01

* = p-value < .05; ns = p-value>.05.

Based on Demographic variables, the H-HD and L-HD groups were different for some features. Specifically, there were significantly more women in the H-HD group than in the other. Moreover, as expected, many H-HD individuals, rather than L-HD ones, reported having a psychological problem and affirmed having an important object they did not wish to discard. Finally, H-HD individuals obtained greater scores at the BAI, the BDI-II, and the OCI-R. Considering the relations between HD, depression, anxiety, and obsessive-compulsive symptoms [21, 54–56], the effect of gender, the BAI, the BDI-II, and the OCI-R total scores were controlled for in the subsequent analyses.

We verified if the H-HD group obtained higher scores on HD-related dysfunctional beliefs, distress tolerance, and emotional dysregulation questionnaires than the Low-HD group.

As shown in Table 2, the H-HD group obtained higher scores for all the measures related to the aforementioned emotional processes, and also concerning the respective subscales. If the effect of gender, the BAI, the BDI-II, and the OCI-R scores were not controlled for, differences between groups were maintained (13.91<F<165.96; p-value < .001).

**Table 2:** Comparisons between the high and the low HD groups (controlling for the effect of gender, the BAI, BDI-II, OCI-R).

	High HD N^j^ = 53	Low HD N^j^ = 55	F _(1, 107)_	Partial Eta squared
SCI^a^	80.81 (22.91)	36.96 (10.42)	47.49***	0.70
M^h^ (DS)^i^				
SCI-EA^a^	31.56 (12.21)	13 (3.79)	33.58***	0.62
M^h^ (DS)^i^				
SCI-C^a^	14.62 (4.47)	8.18 (3.83)	14.21***	0.41
M^h^ (DS)^i^				
SCI-R^a^	18.19 (7.04)	8.73 (4.07)	22.03***	0.52
M^h^ (DS)^i^				
SCI-M^a^	16.17 (6.62)	7.05 (2.62)	21.35***	0.51
M^h^ (DS)^i^				
PERS-GNR^b^	53.42 (9.80)	36.78 (12.73)	26.58***	0.57
M^h^ (DS)^i^				
PERS-GPR^b^	50.83 (10.54)	48.25 (11.50)	6.28***	0.24
M^h^ (DS)^i^				
DERS^c^	101.38 (21.13)	69.20 (17.37)	40.85***	0.67
M^h^ (DS)^i^				
DERS-LA^c^	15.42 (6.12)	9.84 (4.31)	12.82***	0.39
M^h^ (DS)^i^				
DERS-DD^c^	18.13 (4.74)	11.24 (4.38)	19.39***	0.49
M^h^ (DS)^i^				
DERS-C^c^	16.60 (5.34)	9 (3.58)	31.01***	0.60
M^h^ (DS)^i^				
DERS-LC^c^	22.13 (5.52)	15.89 (5.20)	22.89***	0.53
M^h^ (DS)^i^				
DERS-DR^c^	13.89 (3.84)	9.31 (2.88)	21.85***	0.52
M^h^ (DS)^i^				
ASI-3^d^	26.36 (12.28)	11.78 (9.21)	26.23***	0.56
M^h^ (DS)^i^				
ASI-3-PC^d^	7.53 (5.67)	3.55 (4.19)	12.62***	0.38
M^h^ (DS)^i^				
ASI-3-CC^d^	7.81 (5.05)	2.04 (3.38)	22.67***	0.53
M^h^ (DS)^i^				
ASI-3-SC^d^	11.02 (5.08)	6.20 (4.49)	10.78***	0.35
M^h^ (DS)^i^				
DTS^e^	42.94 (10.49)	57.33 (11.75)	30.02***	0.60
M^h^ (DS)^i^				
DTS-T^e^	8.26 (2.72)	11.33 (2.91)	16.46***	0.45
M^h^ (DS)^i^				
DTS-AB^e^	8.87 (2.94)	12.16 (2.90)	24.43***	0.55
M^h^ (DS)^i^				
DTS-R^e^	7.89 (3)	10.07 (3.08)	6.25***	0.23
M^h^ (DS)^i^				
DTS-AP^e^	17.92 (4.49)	23.76 (5.08)	24.17***	0.54
M^h^ (DS)^i^				

Notes

^a^SCI = Saving Cognition Inventory-total score; SCI-ER = Saving Cognition Inventory-Emotional Attachment; SCI-C = Saving Cognition Inventory-Control; SCI-R = Saving Cognition Inventory-Responsability; SCI-M = Saving Cognition Inventory-Memory

^b^PERS-GNR = Perth Emotional Reactivity Scale- General Negative Reactivity; PERS-GPR = Perth Emotional Reactivity Scale- General Positive Reactivity

^c^DERS = Difficulties in Emotion Regulation Strategies- total score; DERS-LA = Difficulties in Emotion Regulation Strategies-Lack of Acceptance; DERS-DD = Difficulties in Emotion Regulation Strategies-Difficulty in Distraction; DERS-C = Difficulties in Emotion Regulation Strategies-Lack of Control; DERS-LC = Difficulties in Emotion Regulation Strategies-Lack of Confidence; DERS-DR = Difficulties in Emotion Regulation Strategies-Difficulty in Recognizing Emotions

^d^ASI-3 = Anxiety Sensitivity Index-3-total score; ASI-3-PC = Anxiety Sensitivity Index-3-Physical Concerns; ASI-3-CC = Anxiety Sensitivity Index-3-Cognitive Concerns; ASI-3-SC = Anxiety Sensitivity Index-3-Social Concerns

^e^DTS = Distress Tolerance Scale-total score; DTS-T = Distress Tolerance Scale-Tolerance; DTS-AB = Distress Tolerance Scale-Absorpion; DTS-R = Distress Tolerance Scale-Regulation; DTS-AP = Distress Tolerance Scale-Appraisal

^h^M = Mean

^i^SD = Standard Deviation

^j^N = size of the group

^k^F = Fisher’s F

*** = p-value < .001

** = p-value < .01

* = p-value < .05; ns = p-value>.05.

### Differences in discomfort related to leaving the object

Before individuals left their object in the laboratory, they had to rate their level of discomfort based on a VAS scale from 0 to 100, where 0 meant “absence of distress and discomfort to leave the object”, and 100 stood for “extreme distress and discomfort”.

The H-HD group obtained, on average, higher scores (M = 65.10; SD = 26.03) than the L-HD one (M = 41.70; SD = 26.15) related to distress/discomfort (F_(1,102)_ = 20.91; p-value < .01).

### Differences in state anxiety and emotions

To investigate the differences between the H-HD and the L-HD groups at T1 and T2, we applied a repeated measures MANOVA. No significant interaction emerged between the “time” and “group” factors for state anxiety (STAI-Y1), positive and negative emotions (PANAS) (F = 1.18; p-value = .32). Significant differences for “time” (F = 4.24; p-value = .007; Partial Eta Squared = 0.11) and “group” (F = 11.60; p-value < .001; Partial Eta Squared = 0.25) have separately emerged. The H-HD group had higher state anxiety levels and greater negative affect than the other group, both at T1 (STAI-Y1: t = 5.07; p-value < .001; PANAS-Negative Affect: t = 5.68; p-value < .001) and T2 (STAI-Y1: t = 4.17; p-value < .001; PANAS-Negative Affect: t = 4.13; p-value < .001), (see Table 3).

**Table 3:** Repeated measures MANOVAs–tests of between-subjects effects.

Dependent Variables	Time	Group type	M^a^ (SD)^b^	F^c^	Partial Eta squared
**STAI-Y1**	**T1**	High HD	42.83 (10.75)	26.22***	0.20
		Low HD	33.07 (9.22)		
	**T2**	High HD	41.45 (10.61)		
		Low HD	33.09 (10.23)		
**PANASNegative Affects**	**T1**	High HD	19.70 (6.90)	32.20***	0.23
		Low HD	13.22 (4.81)		
	**T2**	High HD	17.08 (6.84)		
		Low HD	12.65 (3.96)		

Notes

^a^M = Mean

^b^SD = Standard Deviation

^c^F = Fisher’s F

*** = p-value < .001

** = p-value < .01

* = p-value < .05; ns = p-value>.05.

A significant difference between T1 and T2 emerged for the PANAS-Negative Affect subscale (F = 8.44; p-value < .01; Partial Eta Squared = 0.07), showing a decrease in negative emotions, independent of the effect of "group”. Moreover, again examining the within-subject effects, a slight increase of positive emotions between T1 (M = 27.35; SD = 8.42) and T2 (M = 28.69; SD = 8.72) has been highlighted (F = 4.73; p-value < .05; Partial Eta Squared = 0.04).

To investigate whether the decrease of negative emotions over time concerned one or both groups analyzed separately, we ran a Paired-Samples T-test for the PANAS scales. A significant difference was found for negative emotions between T1 and T2 for the H-HD group (PANAS-Negative Affect: t = 2.92; p-value < .01), indicating more negative emotions when individuals with hoarding features had to leave the object (T1) (H-HD: M = 19.70, SD = 6.90) as compared with their emotions in T2 (M = 17.08, SD = 6.84).

### Frequency of thoughts and feelings during the week

To investigate differences in the frequency of negative emotions that participants referred to each day during the week, we applied repeated measures MANOVA considering the frequency of thoughts and feelings as dependent variables, as well as the object-related level of distress reported in the Daily self-monitoring schedule.

No significant interaction emerged between the “time” and “group” factors (F = 1.53; p-value >.05). Significant differences for “time” (F = 6.60; p < .01; Partial Eta Squared = 0.89) and “group” (F = 17.47; p < .001; Partial Eta Squared = 0.61) have separately emerged.

**Fig 2:**
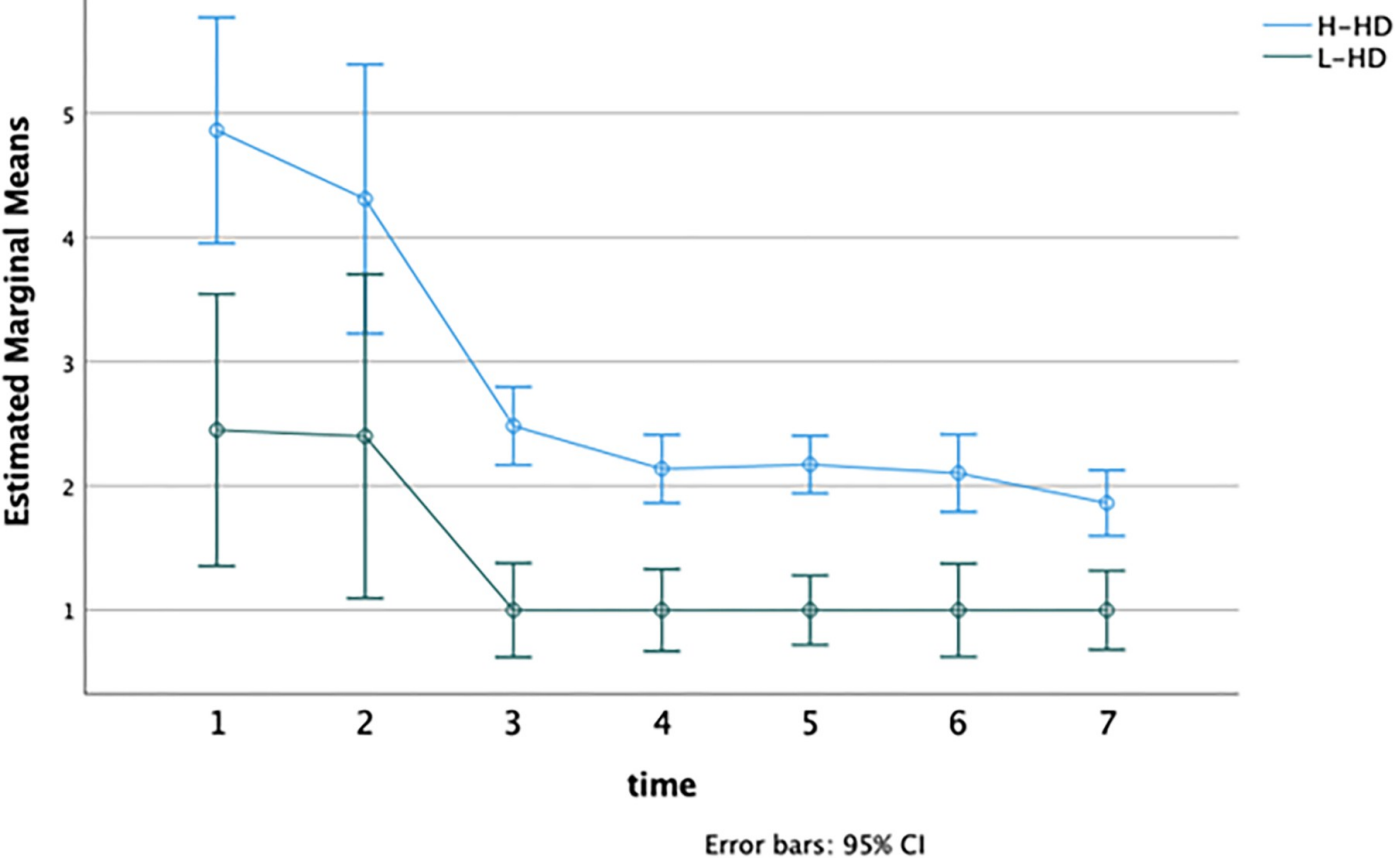
Frequency of thoughts object-related referred each day respectively for H-HD and L-HD. **Notes**: H-HD: group of individuals with higher hoarding features; L-HD: group of individuals without hoarding features; Time 1–7: number of the day.

**Fig 3:**
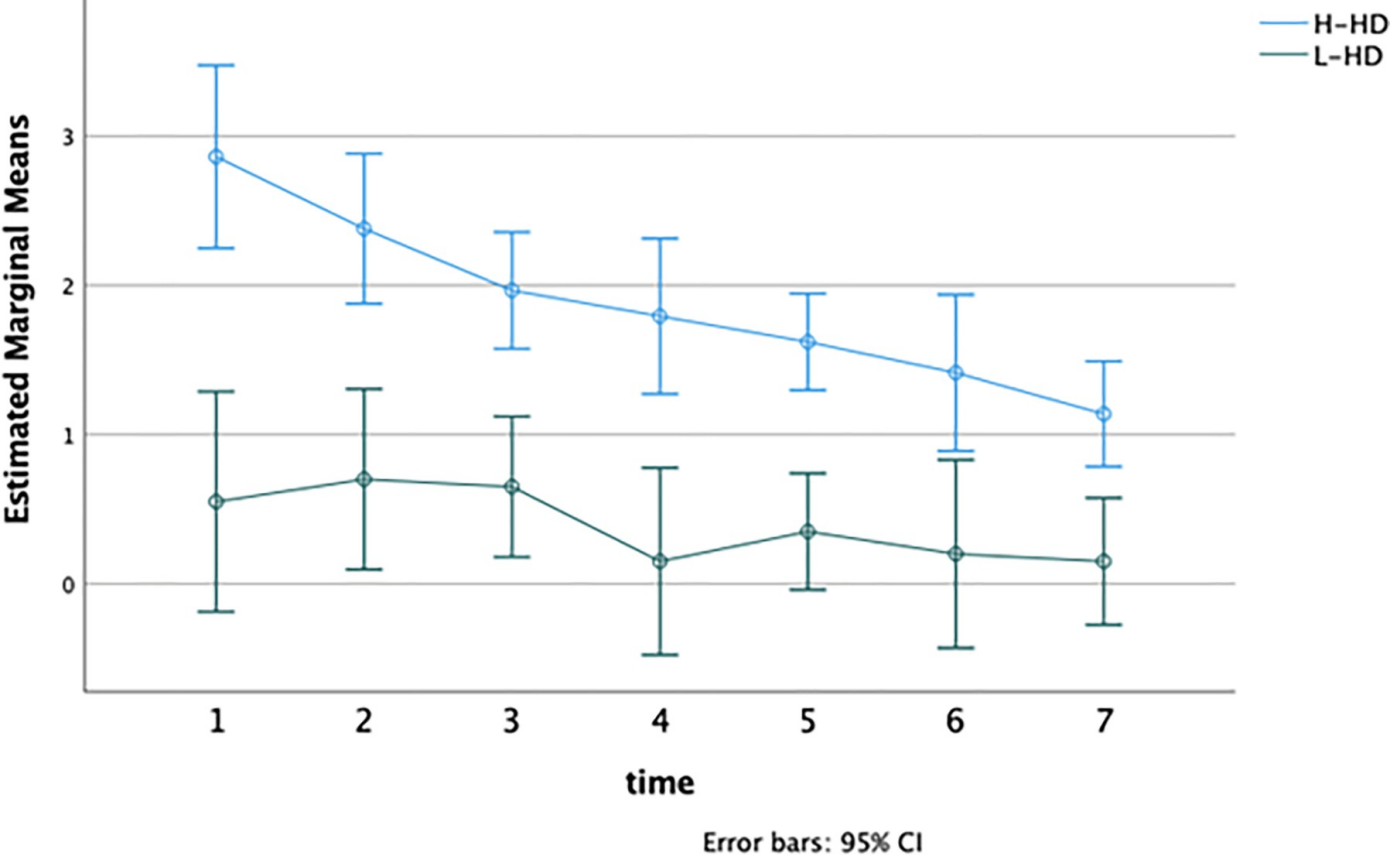
Difference in the frequency of negative emotions object-related referred each day. **Notes**: H-HD: group of individuals with higher hoarding features; L-HD: group of individuals without hoarding features; Time 1–7: number of the day.

**Fig 4:**
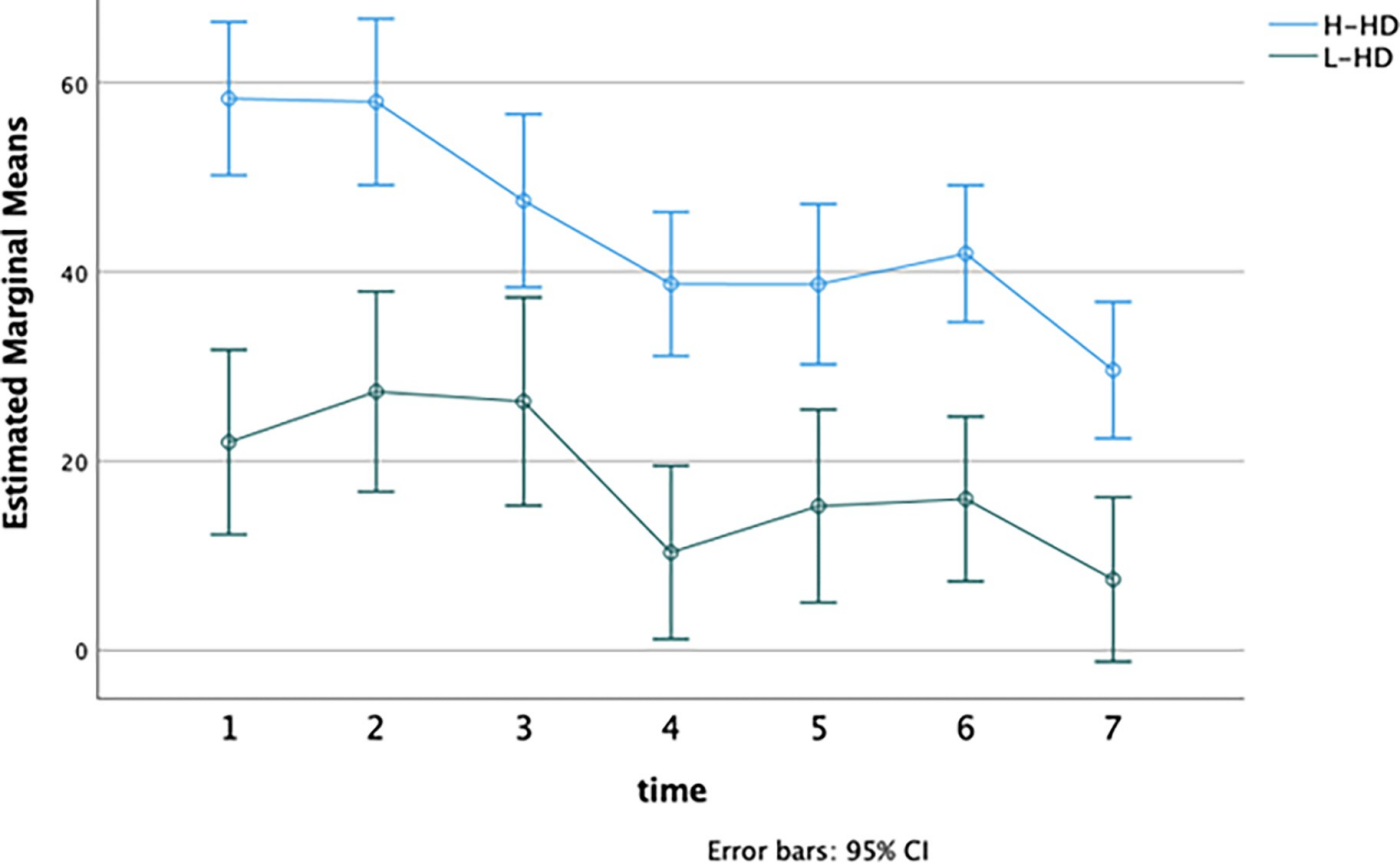
Difference in the level of distress object-related referred each day. **Notes**: H-HD: group of individuals with higher hoarding features; L-HD: group of individuals without hoarding features; Time 1–7: number of the day.

As expected, H-HD individuals had more frequent thoughts about their objects during the week as compared with the other group (H-HD: M = 12.25, DS = 6.61; L-HD: M = 6.96, DS = 5.19; F = 27.33, p-value < .001) (Fig 2). The H-HD group referred to a total of 654 beliefs related to the object (62.23%), against 397 of the L-HD (37.8%). Moreover, each day, the H-HD group reported a greater frequency of negative emotions (F = 35.72, p-value < .001) and higher levels of distress than the L-HD group (F = 46.59, p-value < .001) (Figs 3 and 4).

Globally, the H-HD group referred to 561 negative emotions related to the object (70.13%), against 239 of the L-HD (29.9%). As we already mentioned, it is essential to highlight that the frequency of negative emotions and dysfunctional beliefs decreased during the week for both groups separately (Figs 2 and 3).

Overall, we identified five key themes in the thoughts that participants expressed during the week: 1) “no worries about the object”; 2) “feeling the lack of the object”; 3) “concern about where the object is and that something bad might happen to the object”; 4) “impatience to get the object back”; 5) “feeling of insecurity in being without the object”.

**Fig 5:**
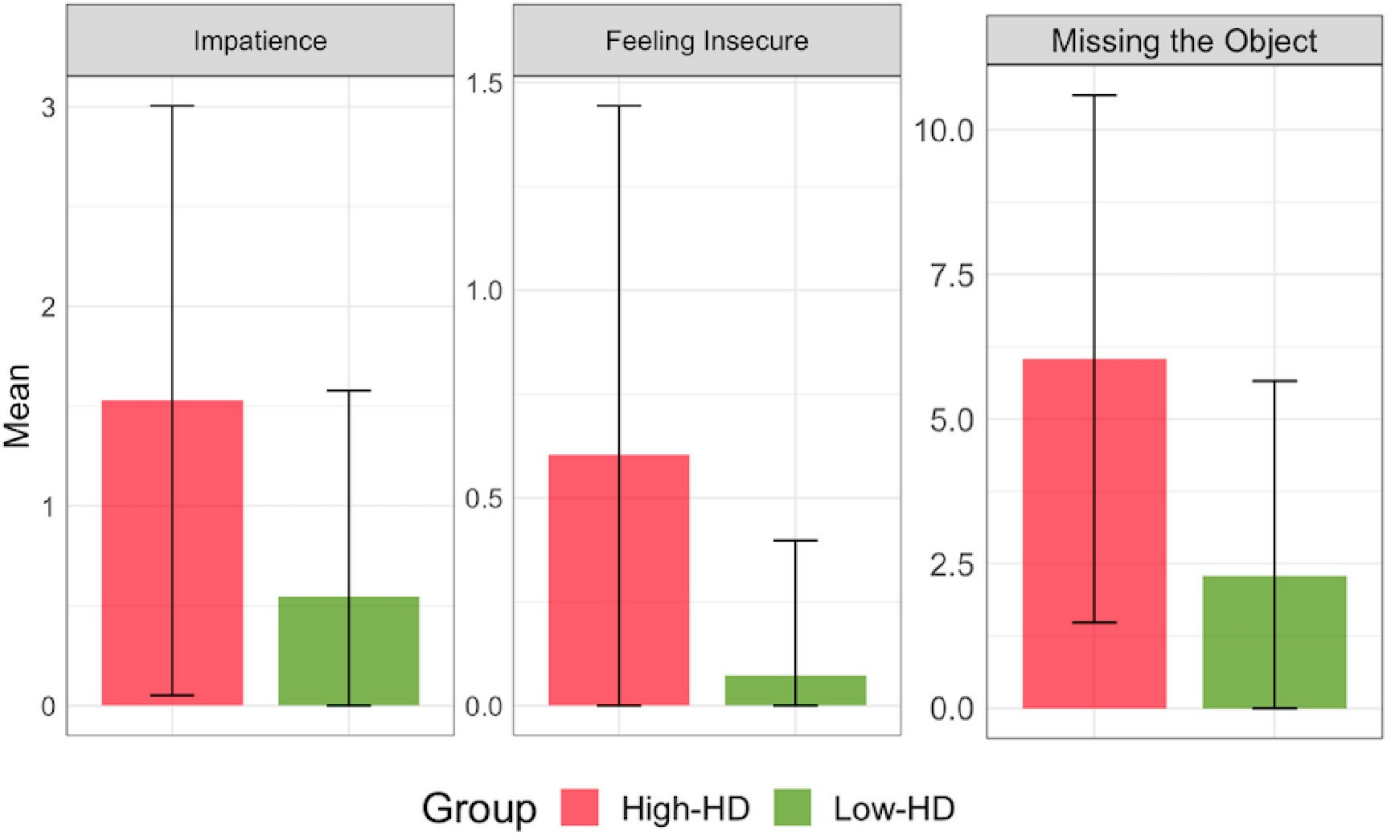
Differences between groups in the types of thoughts reported during the week.

For each theme, a comparison between the two groups was calculated. Specifically, statistically significant differences between the groups emerged for “feeling the lack of the object (Missing the Object)” (H-HD: M = 6.04, DS = 4.56; L-HD: M = 2.29, DS = 3.37; F(1,107) = 23.72, p-value < .001), “impatience to get the object back (Impatience)” (H-HD: M = 1.53, DS = 1.48; L-HD: M = 0.55, DS = 1.03; F(1,107) = 16.18, p-value < .001), and “feeling of insecurity in being without the object (Feeling Insecure)” (H-HD: M = 0.60, DS = 0.84; L-HD: M = 0.07, DS = 0.33; F(1,107) = 19.03, p-value < .001), (see Fig 5).

Additionally, from a qualitative perspective, some participants added interesting insights, referring to progressively becoming accustomed to being without their objects and, although at the beginning they thought about it a lot, towards the end they felt they could do without it. For example, on the first day, a woman assumed that her object brought her luck and she felt extremely insecure without it. As the days passed, however, she began to notice how she managed to face some events even without her object; this made her think about the actual value of the possession and the possibility of getting rid of it without problems if she wanted to.

### Linear regression analysis

A linear regression analysis based on the stepwise method was conducted on the entire sample to investigate whether dysfunctional thoughts, difficulty in tolerating distress, and emotional dysregulation were predictive of the frequency of thoughts and negative emotions, the intensity of the emotions, and the discomfort experienced when participants had to leave their object in the laboratory.

We considered the SCI (Saving Cognitions Scale), the DERS (Difficulties in Emotion Regulation Scale), the ASI-3 (Anxiety Sensitivity Scale), the DTS (Distress Tolerance Scale), and the PERS-General Negative Reactivity as predictors, and the frequency of thoughts expressed during the week about the object, the frequency of negative emotions of the week, and the intensity and discomfort related to leave the object as dependent variables.

Data showed that frequencies of thoughts, negative emotions, and intensity of emotions were related to the SCI total score (Adjusted R Square = 0.28; F_(5,107)_ = 9.13; p-value < .001; Adjusted R Square = 0.18; F_(5,107)_ = 5.80; p-value < .001; Adjusted R Square = 0.25; F_(5,107)_ = 7.65; p-value < .001), (see Table 4).

**Table 4:** Linear regression models considering the frequency of thoughts and negative emotions, and the intensity of emotions related to the object expressed during the week.

Predictors	Frequency of thoughts	Frequency of negative emotions	Intensity of emotions
	β (t)	β (t)	β (t)
**SCI**	**.52 (4.67** *** **)**	**.52 (2.89** ** **)**	**.43 (3.74** *** **)**
**DERS**	-.09 (.62)	-.09 (0.85)	.15 (0.92)
**ASI-3**	-.50 (-1.19)	-.50 (-0.31)	-.05 (-0.41)
**DTS**	-.57 (0.15)	-.57 (0.52)	.003 (0.2)
**PERS-GNR**	-.01 (.81)	-.01 (1.01)	.07 (0.44)

Notes

SCI = Saving Cognition Inventory-total score; DERS = Difficulties in Emotion Regulation Strategies-total score; ASI-3 = Anxiety Sensitivity Index-3-total score; DTS = Distress Tolerance Scale-total score; PERS-GNR = Perth Emotional Reactivity Scale-General Negative Reactivity

*** = p-value < .001

** = p-value < .01

* = p-value < .05.

Data evidenced that not only beliefs about leaving a personal object (SCI), but also sensitivity to anxiety (ASI-3), and distress tolerance (DTS) were predictive of the experienced discomfort only when participants of the H-HD group had to decide to leave their objects in the laboratory (Adjusted R Square = 0.14; F_(5,52)_ = 2.71; p-value = .03) (see Table 5).

**Table 5:** Linear regression models for the self-report questionnaires and the discomfort of leaving the object (T1), considering the high-HD group.

Predictors	Discomfort to leave the object
	β (t)
**SCI**	.08 (1.004*)
**DERS**	-.09 (-0.84)
**ASI-3**	-.50 (-2.88**)
**DTS**	-.57 (-2.45*)
**PERS-GNR**	-.01 (-0.03)

Notes

SCI = Saving Cognition Inventory-total score; DERS = Difficulties in Emotion Regulation Strategies-total score; ASI-3 = Anxiety Sensitivity Index-3-total score; DTS = Distress Tolerance Scale-total score; PERS-GNR = Perth Emotional Reactivity Scale-General Negative Reactivity

*** = p-value < .001

** = p-value < .01

* = p-value < .05.

## Discussion

Our study’s aim was to investigate the relationship between HD symptoms and the transdiagnostic constructs related to emotional processes that are relevant to the hoarding framework.

People in the H-HD group obtained higher scores on questionnaires investigating dysfunctional beliefs related to possessions, anxiety sensitivity, distress tolerance, and emotional dysregulation when they were asked to leave their object at the laboratory. These results aligned with previous studies which showed that individuals with hoarding features have more difficulty regulating emotions and tolerating distress (e.g., the distress experienced when they have to discard an object) than individuals without hoarding features and healthy controls [6–24]. Indeed, PwH have been shown to exhibit less emotional clarity, an unwillingness to accept certain emotions, and struggle with regulating emotions and remaining involved in goal-directed behavior [13, 21].

We also aimed to explore the level of discomfort that participants felt when they had to leave their objects at the laboratory. We found that H-HD participants experienced greater discomfort levels than L-HD individuals prior to leaving their objects. This is in line with another study which found that distress levels before and during a discarding task were higher in the HD group [24]. Our data showed that having dysfunctional beliefs related to one’s possessions, high anxiety sensitivity, and difficulties tolerating distress all contribute to explain why people with H-HD felt greater levels of discomfort when they had to separate from a valuable object. This data highlights the importance of allowing PwH in improving their emotional regulation abilities before discarding their possessions.

We expected that individuals, especially those in the H-HD group, would report higher state anxiety levels and more negative emotions when they left the possession at the laboratory (T1) compared to when they received the object back (T2). Our results supported these hypotheses as shown by the differences that emerged between the H-HD and L-HD groups for state anxiety and negative emotions both at T1 and T2. Considering the H-HD group has higher scores in questionnaires investigating anxiety and depression, the differences that emerged between the two groups for state anxiety and negative emotions, both in T1 and T2, were in line with our hypotheses. However, the two groups did not differ in the intensity of anxiety and negative emotions experienced over time. This can be due to the fact that our sample did not comprise diagnosed individuals.

The main finding in this study was that only those with hoarding characteristics experienced a greater intensity in their negative emotions when leaving their object (T1) compared to when they took it back (T2). This data aligns with the conceptualization of the cognitive and behavioral model of hoarding [3]. Differences from T1 to T2, and the progressive increase of positive emotions throughout the week, could lead to the hypothesis that approaching the timepoint at which one knows that their object will be returned to them contributes to the reduction of negative feelings. However, the general gradual reduction of the frequency of thoughts, negative emotions, and object-related distress that developed over the week also suggests that habituation played a role [57]. Indeed, a week spent without using peculiar techniques to manage object-related distress may have reduced the intensity of negative emotions that were more frequently elicited when the participants were directly exposed to separate themselves from possessions. The hypothesis that habituation was involved is also in line with some cognitive and behavioral techniques that exist to reduce anxiety, rumination, worry, and obsessive thoughts. This progressive reduction in thoughts frequency and negative emotions was evident in participants’ daily schedule reports. For example, especially at the beginning of the week, some participants attributed adverse events that took place on those days to being separated from their possession. As the week progressed, however, participants acknowledged that this was not accurate, therefore affirming that the time that someone who hoards spends without a possession helps them to understand that their beliefs influence their feelings. Even though participants from the H-HD group still thought a lot about their object during the week, they gradually became more accustomed to being without their possession, which they thought would be an impossible feat at the beginning of the week. Ultimately, the detachment from an object helped participants to develop an understanding that they are capable of living without it. This is a crucial insight that informs one of the first activity of the treatment for HD. Indeed, a patient’s awareness of their symptoms and motivation to adhere to treatment is necessary for cognitive and behavioral techniques to be effective, such as the rehabilitation of cognitive functions (e.g., the planning and organization of objects and spaces of the house or the disposal of their assets, and so forth), and exposure therapy [58].

In addition, some individuals have gradually become accustomed to being without their belongings, although they have thought about it a lot and would never have imagined being able to detach from them at the beginning of the week.

Data show the prominent role of cognitive processes in maintaining and regulating emotions and their influence in promoting awareness and adherence to the treatment, other than facilitating the possibility to discard objects, promoting the development of valuable skills during, for example, the act of discarding [6].

Consistent with Frost et al. [3] and Phung et al. [6], our findings support the theory behind HD and have clinical implications. Indeed, both qualitative and quantitative data indicate that the difficulty of people with HD features to tolerate and regulate emotions may be a result of them dysfunctionally believing the object to be helpful in obtaining something, feeling safe, or being happy. This act as both a risk and a maintenance factor for hoarding behaviors. When these individuals are exposed to anxiety-related physiological responses and attribute a special power to the object of regulating their emotions they are likely to avoid throwing it away. Moreover, the perception of high negative emotion level intensity, which arises from a greater sensitivity to anxiety, may play a role in reinforcing the value that PwH attributes to an object, making discarding even more difficult.

As highlighted by Grisham et al. [59], our findings suggest and support the importance of including specific sessions in HD treatment to assessing and managing the patients’ difficulties in tolerating and regulating emotions. This is crucial, especially considering the virtuous effect transdiagnostic constructs can have in working on HD symptoms, with a pervasive impact on the well-being of PwH and their families.

Since our study also exemplified how difficult it is for PwH to discard their possessions, as shown by the presence of intrusive thought and negative emotions that PwH reported even when they knew that their object was being kept safe in the laboratory, we suggest adding a new intermediate phase to HD treatment. In this phase, the patient should be asked to temporarily separate from their possession before discarding them. Introducing this phase will allow the PwH to gradually work through their negative emotions and dysfunctional beliefs and give them more time to process the detachment from their object. Introducing this phase may help to reduce the possibility of avoidance behaviors and treatment dropout.

Our findings may be used to educate public agencies about effective ways to help PwH. Currently, public services mostly respond to PwH by going to their place of residence and discarding almost all of their possessions. However, clearing activities tend to be ineffective in helping PwH overcome their disorder and may actually increase its severity. Indeed, public services that involve clearing activities often do not provide PwH with strategies to manage their disorder. In addition, clearing activities may involve exposing PwH to a situation that is too intense if exposure sessions in a treatment setting was not conducted first. Developing an effective treatment that considers all the cognitive, behavioral, and emotional factors involved in HD may be of special interest to public agencies since eviction and homelessness is relatively common among PwH. Indeed, in a study with 864 PwH, 2% reported being evicted, and 6% reported being threatened with eviction [60]. Moreover, another study with 78 randomly selected homelessness individuals showed that up to 21% of participants had HD symptoms and 8% affirmed that these symptoms contributed to their homelessness condition [61].

It is also important to consider that PwH often do not seek mental health treatment, but they first come to the attention of non-mental health agencies during emergencies (e.g., fire) [62–64]. If they received psychiatric or psychological treatment (48%), they would be more likely to be treated for comorbid psychiatric diagnoses, such as major depressive disorder [64, 65]. Therefore, working on emotion regulation strategies before exposing the person to discarding the object and improving skills associated with managing emotions is essential. This research also intends to raise awareness of HD as a condition frequently related to a psychological disorder requiring specific treatment.

The current study has some limitations. Our sample was mostly comprised of university students, which limits the generalizability of our results. As such, future research should follow the current study procedure using a more heterogeneous sample of individuals, including people with HD and a wide range of frequently comorbid psychopathologies and sociodemographic features that are critical for HD [e.g., 66–68].

Future studies may also investigate other constructs related to hoarding, such as an intolerance of uncertainty and negative urgency, which are related to difficulties in tolerating distress and regulating emotions [e.g., 6, 69]. The introduction of a control group comprising participants who do not have to hand over their possession could also be a useful comparison.

The current contribution is intended to examine how extremely high and extremely low traits of hoarding features unfold in a non-clinical sample, considering that it is not possible to diagnose a mental disorder using only a single self-report measure. This was helpful in better highlighting the differences between a sample extracted from the general population with high and low hoarding features. Maximizing variance in non-clinical samples allowed us to better characterize hoarding better and to pinpoint its transdiagnostic features, allowing for the application of this research model on clinical samples in the future. Finally, based on a more ecological perspective, intermediate hoarding scores should be obtained since it would be helpful to deeply understand the way that individuals with non-extreme scores in the SI-R relate to their possessions. For this reason, future studies aiming to replicate this research should consider including an additional group with moderate hoarding symptoms and adjusting the design to accommodate hoarding intensity as a continuous variable.

## Conclusions

In this longitudinal study, we investigated the emotional and cognitive components of HD when individuals with H-HD and L-HD were separated from their possessions. We found that individuals with hoarding features experienced difficulty throughout the week after they were separated from a possession, even when they did not have diagnosed HD and knew that they would be receiving their object back soon. Integrating our proposed intermediate phase to standard HD treatment may be helpful in allowing individuals more time to process being detached from their possession, and therefore ultimately reduce the possibility of avoidance behaviors and treatment dropout. Overall, our findings confirmed the critical role of other transdiagnostic constructs on hoarding features and underscore the importance of considering them during HD assessment and treatment.

## Supporting information

S1 File Highlights (bullet points useful in capturing the main results of the study). (DOCX)
