# Supplementary material for: The experience of leaving a valuable object: An investigation of emotional processes related to Hoarding disorder features
Source: PLoS One. 2023 Feb 14;18(2):e0280933. doi: 10.1371/journal.pone.0280933 (PMC9928071; doi:10.1371/journal.pone.0280933)
Supplement: S1 File — (DOCX) [file pone.0280933.s001.docx]

**Highlights:**

- Cross-sectional and longitudinal investigation of the relation between hoarding features and emotional processes.
- No studies have investigated the frequency and intensity of emotions during a period without an important object.
- At T0, the H-HD individuals showed higher levels of anxiety sensitivity, distress tolerance, and emotional dysregulation rather than the L-HD.
- H-HD subjects only referred to more negative emotions when they had to leave a personal object in the lab rather than when they have to take it back.
- H-HD individuals have more intrusive beliefs-object related and experience a major frequency of negative emotions during the week than Low-HD individuals.
- The SCI total score is related with the frequencies of thoughts and negative emotions reported during the week.
- Dysfunctional beliefs, anxiety sensitivity and difficulty to tolerate distress are in relation with discomfort related to leaving the object.
